# Towards an Effective, Rational and Sustainable Approach for the Control of Cattle Ticks in the Neotropics

**DOI:** 10.3390/vaccines8010009

**Published:** 2019-12-30

**Authors:** Agustín Estrada-Peña, Matías Szabó, Marcelo Labruna, Juan Mosqueda, Octavio Merino, Evelina Tarragona, José M. Venzal, José de la Fuente

**Affiliations:** 1 Department of Animal Pathology, Faculty of Veterinary Medicine, 50013 Zaragoza, Spain; 2 Research Group in Emerging Zoonoses, IA2, 50013 Zaragoza, Spain; 3 Federal University of Überlandia, Überlandia 38408-100, Brazil; szabo@famev.ufu.br; 4 University of Sao Paulo, Sao Paulo 05508-220, Brazil; labruna@usp.br; 5 Faculty of Veterinary Medicine, Autonomus University of Querétaro, Santiago de Querétaro 76010, Mexico; joel.mosqueda@uaq.mx; 6 Faculty of Veterinary Medicine, University of Tamaulipas, Tamaulipas 87000, Mexico; mero840125@hotmail.com; 7 INTA Rafaela, Santa Fe RN34 227, Argentina; tarragona.evelina@inta.gob.ar; 8 Faculty of Veterinary Medicine, University of the Republic, Salto 11200, Uruguay; dpvuru@hotmail.com; 9 SaBio, Instituto de Investigación en Recursos Cinegéticos (IREC-CSIC-UCLM-JCCM), Ronda de Toledo s/n, 13005 Ciudad Real, Spain; jose_delafuente@yahoo.com; 10 Department of Veterinary Pathobiology, Center for Veterinary Health Sciences, Oklahoma State University, Stillwater, OK 74078, USA

**Keywords:** tick, vaccine, ecology, neotropics, cattle, wildlife, pathogen

## Abstract

Ticks and transmitted pathogens constitute a major burden for cattle industry in the Neotropics. To address this limitation, the Spanish Ibero-American Program of Science and Technology in Development office (CYTED) supported from 2018 a network of scientists named “LaGar” (CYTED code 118RT0542) aimed at optimizing the control strategies of cattle ticks in the neotropical region. As part of network activities, a meeting and course were organized on 4–8 November 2019 in Querétaro, Mexico to address the objective of developing the infrastructure necessary for an effective, sustainable (i.e., combination of efficacious acaricides with anti-tick vaccines) and rational (i.e., considering tick ecology, seasonal dynamics and cattle-wildlife interactions) control of cattle tick infestations and transmitted pathogens. The course was focused on scientists, students, cattle holders and producers and pharmaceutical/industry representatives. In this way the course addressed the different views presented by participants with the conclusion of producing a research-driven combination of different interventions for the control of tick tick-borne diseases.

## 1. Introduction: The Need to Optimize Control Strategies of Ticks and Tick-Borne Pathogens Affecting Cattle in the Neotropics

Ticks and tick-borne pathogens, like protozoans of the genus *Babesia*, and bacteria of the genus *Anaplasma* are among the most important pests of cattle in Central and South America, as well as in many other parts of the world. Most of the estimated world’s population of 1.2 billion cattle are at risk of exposure to tick and tick-transmitted pathogens, producing significant losses derived of decreased meat and milk production, or fatalities [1,2]. The bovine babesiosis (also known as cattle fever and transmitted by ticks of the subgenus *Boophilus*) is still seriously affecting cattle production in wide regions of the world. The disease is produced by several species of protozoans, of which most important are *Babesia bovis* and *B. bigemina*, which are transmitted by *Rhipicephalus (Boophilus) microplus* and *R. annulatus* among cattle and other wild ungulates. The disease is difficult to detect in chronically infected animals that serve as reservoirs: ticks ingest the parasite with blood meals and efficiently transmit to naïve cattle [3]. *Rhipicephalus* ticks and the pathogens they transmit present significant threats to cattle populations worldwide. While *R. annulatus* is restricted to some parts of Mexico and USA, *R. microplus* is a successful species that extend from the USA-Mexico border to northern Argentina. To these two species, it is necessary to add the species in the group *Amblyomma cajennense*, which have in most cases a great interest in human health, but that also have an impact on animal production.

Control measures for tick-borne pathogens have commonly focused on the tick vectors, ignoring the large interactions of the ticks and the environment. The holistic view of tick-host-environment is capital to continued efficacy of control measures [4,5,6,7]. Pioneering studies addressed the investigation of tick population dynamics, interactions with livestock and wild hosts, and tick’s seasonal activity that is driven by climate factors [6,7,8,9,10,11,12,13,14]. The development of models explaining all these traits helped to understand both the spatial dynamics and the climate suitability of pathogen-transmitting ticks, [11,12,15]. However, the control of tick-transmitted pathogens still needs a detailed regional-scale spatial modelling framework identifying environmental conditions and landscape patterns driving to the establishment and spread of permanent cycles of transmission.

The control of cattle ticks infestations in the neotropics is still dependent on the generalized use of classic acaricides without strategies considered as tick ecology, the possible endemic stability of tick-borne pathogens, its economic impact, or the importance of other vertebrates in the spread of these pathogens. This is causing the emergence of multiacaricide-resistant ticks with a large impact on cattle production [7,8,9,10]. The resistance of ticks to acaricides is widespread. The Food and Agriculture Organization of the United Nations (FAO), recently proposed an agenda to activate a coordinated program of tick control in Central and South America. The FAO working group on tick resistance to ixodicides, together with the coordinated inputs by major pharmaceutical companies proposed measures to address that effort, including: (i) the correct application of the acaricides, including considerations of efficacy and efficiency; (ii) the development of nationwide community and farmer sensitization programs on ticks; (iii) the continuous monitoring of tick populations, the inter-year changes of pathogens prevalence, and the emergence of resistance to acaricides; (iv) the monitoring of wildlife-tick-livestock interactions; and (v) the development of new anti-tick vaccines.

In this context, the Spanish CYTED office approved in 2018 and continues supporting a network of scientists named “LaGar” (CYTED code 118RT0542) aimed to optimize the control strategies of ticks in the neotropical region. The objectives of the network are to elaborate on a solid framework aimed to optimize the methods for ticks control, pivoting over three basic strategies, namely (i) the ecology of the ticks; (ii) the interface of the tick-cattle-wild animals interface and the ecological studies aimed to disentangle this complex system; and (iii) the optimization of the use of classic acaricides and novel vaccine interventions.

## 2. The Course: Building Scientific Capacity in Mexico

As part of the activities of the network, a course was organized in 4–8 November 2019 to address the objective of developing the infrastructure necessary to understand how the climate may alter the finely tuned patterns of tick activity, the changing patterns of infections by tick-borne pathogens, the importance of the wildlife as super-spreaders of ticks vectors and pathogens, and the role of vaccines in the control of ticks.

**Figure 1:**
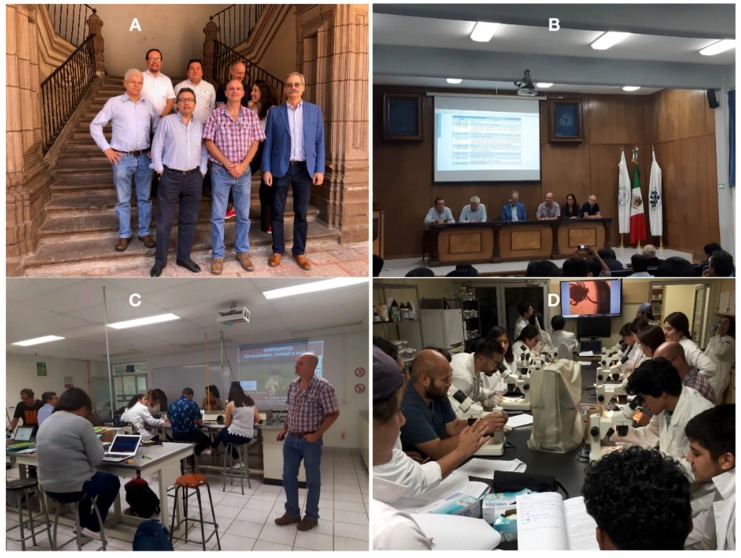
Some events in the meeting of the network “LaGar” in Querétaro, México. (**A**) The members of the network who attended the meeting. From left to right, top row, Juan Mosqueda, Octavio Merino, and Matias Szabó; mid row, Marcelo Labruna and Evelina Tarragona; bottom row, Agustín Estrada-Peña, José M. Venzal, and José de la Fuente. (**B**) The main session of lectures on tick ecology and control. (**C**,**D**) Two aspects of the seminar and working sessions on “update of identification of ticks in the Neotropics” lead by J. M. Venzal and E. Tarragona.

The course was intended to disseminate the scientific knowledge in Mexico at both theoretical and laboratory and field practical levels and to implement a multidisciplinary approach for the control of cattle tick infestations and tick-borne infectious diseases affecting humans and animals in Central and South America (Figure 1). A special emphasis was given to the importance of wildlife in the support of permanent populations of ticks affecting cattle, the ecology of ticks, and other factors affecting human and animal health and livestock production and trade.

The program included (i) an introduction to the ecology of ticks in the neotropics in the cattle-wildlife interface by M. Labruna and M. Szabó; (ii) an overview of the distribution and seasonal dynamics of the tick subgenus *Boophilus*, by A. Estrada-Peña; (iii) the current concepts of vaccinomics by J. de la Fuente; (iv) a view to the world of ticks by J.M. Venzal, which was completed by a one-day course capacitation on morphological determination of ticks of importance in the Neotropics, by E. Tarragona and J.M. Venzal. The course was developed in the Faculty of Veterinary Medicine in the University of Querétaro (México) with the joint participation of the National Council of Science of Mexico (CONACYT), The Mexican Service of Animal Health (SENASICA), the World Organisation for Animal Health (OIE) and the Council of Science and Technology of Querétaro, México (CONCYTEQ).

## 3. The Ecology of Ticks as a Framework for Successful and Sustainable Tick Control

The protozoans *B. bovis* and *B. bigemina* are the etiological agents of what is considered “the most economically significant diseases of cattle in tropical and subtropical areas” [15]. All species of the genus *Babesia* are transmitted by ticks that feed on a limited range of hosts. The main vectors of these pathogens are one-host ticks of the genus *Rhipicephalus* spp. ticks, which are widespread in tropical and subtropical countries [1,16]. The exploit of the highly efficient one-host cycle by the ticks allows the quick spread of foci of diseases. These two tick species, once primarily parasites of wild ungulates are the only vectors involved in the biological transmission of babesiosis. The most common incubation period is about 2–3 weeks after tick infestation. The experimental inoculation of *Babesia* commonly produces shorter incubation periods [17]. The clinical manifestations of babesiosis are typical of a haemolytic anemia but vary according to agent and host factors, like age and immune status [17]. After a prepatent period following the onset of feeding by *Babesia*-infected tick larvae, peak parasitemia and the manifestation of clinical signs occur. *Babesia bigemina* matures approximately 9 days after larval attachment, and it is only transmitted by nymphs and adults of the tick [16,18,19,20,21,22].

*Anaplasma marginale* is the most prevalent tick-borne pathogen of cattle worldwide, with endemic regions in North, Central, and South America, as well as Africa, Asia and Australia [23]. Ixodid ticks are the biological vectors of *A. marginale*, while mechanical transmission can occur through fly bites and reuse of needles [5]. The one-host tick *R. microplus* is estimated to be the main vector of *A. marginale* in Brazil [24,25]. Once an animal is exposed to this pathogen, acute infection develops, which is characterized by fever, high levels of bacteremia, anemia, weakness, reduced growth and milk production, miscarriage, and in some cases death [26].

It is well known that any successful tick control framework must to be built over a solid knowledge of the biology of the targeted tick species. This includes not only a reliable knowledge of its distribution, but also the factors behind the realized range of the species, which are dependent on abiotic traits (i.e., temperature and relative humidity) and biotic ones (i.e., hosts). The finely tuned combination of these traits determines the probability a tick can survive in the environment. Human actions on the environment, like populating large areas with livestock, increment the suitability of a habitat for ticks because the high density of hosts.

The increase in development and application of modelling exercises for understanding tick population dynamics had led to the capture of the actions of climate on the basic traits of the performance of tick’s life cycle [12,13,14]. It is widely accepted that vertebrates move through a matrix of suitable habitat, being these movements governed by simple rules depending on the behaviour of the vertebrate species and the habitat/non-habitat geographical patterns [27,28,29]. Studies exist about the importance of the landscape composition on the movement of animals through ‘‘corridors’’ that connect patches of suitable habitat, therefore impacting the abundance of ticks [12]. These movements affect the availability of hosts for ticks at specific points of the habitat [30], thus impacting also tick abundance. The simulation of animal movements, and their preferences towards some types of vegetation, together with the response of ticks to climate has surfaced as a suitable way to capture basic parameters of the phenology and abundance of ticks [19,31].

Further on the spatial scale of tick distribution, there is also a temporal pattern, which draws what is known as “seasonality” or the moments of the year in which ticks are active and questing for hosts. The root of the tick control using classic ixodicides is to know the onset of such period of activity, to match the application of the chemical with the activity of the tick. In some cases, like the ticks of the complex *A. cajennense*, this is further complicated because the immature stages are commonly parasites of small mammals, adults feeding on livestock. The feeding on rodents introduces a potential “noise” because the natural variability of the populations of rodents, and how the different stages of ticks feed on them. To date, efforts to model the seasonality of ticks have been reduced to one host species, like *R. microplus* and *R. annulatus*, the former being most important tick in the region. Efforts have been made using process-driven models [7,14] but a new approach that use direct recordings by satellite imagery is being introduced. The technique is based on the annual variation of both the ground surface temperature and the vegetal vigour to capture the pattern of larval seasonality. Preliminary results support the hypothesis that this method, supported by previous empirical data on tick activation by prevailing weather conditions, will provide an adequate information for predictive mapping of larval *R. microplus* activity.

## 4. The Wild Animals and the Cattle-Tick Interface

Basic to host tick parasitism at the cattle-wildlife interface is the knowledge that much of the existing tick-host association patterns are driven by biogeography and ecological specificity of the parasite [32]. This pattern was reaffirmed for neotropical ticks [33,34] who observed on a meta-analysis that strict host specificity is not common among Neotropical hard ticks and that tick distribution is rather driven by tick ecology and evolution of habitat specificity.

It is important to highlight that cattle raising is necessarily related to a radical environmental alteration: the replacement of native vegetation by pastures. This non-indigenous and artificial environment coupled with high animal density supports high infestations levels of another exotic being, *Rhipicephalus microplus*, a tick species introduced from tropical and subtropical Asia [35] and that became the main cattle tick in the region. Wildlife hosts that regularly or occasionally use pastures may be exposed to exotic *R. microplus* and associated pathogens such as *Babesia* and *Anaplasma* but also other less known as the Mogiana tick virus [36]. Indeed, fully engorged *R. microplus* female ticks are frequently found on hosts such as *Puma concolor* (cougar), *Cerdocyon thous* (crab-eating fox), *Odocoileus virginianus* (White-tailed deer), *Blastocerus dichotomus* (marsh deer) *Ozotoceros bezoarticus* (Pampas deer), *Myrmecophaga tridactyla* (giant anteater) and others [37,38,39,40,41]. The proximity to native fauna and its environment, exposes bovines to indigenous ticks and several were already registered on this host. Albeit in different geographical and ecological settings throughout the Neotropics, species such as *Amblyomma mixtum*, *Amblyomma neumanni*, *Amblyomma parvum*, *Amblyomma sculptum*, *Amblyomma tonelliae*, *Amblyomma triste*, *Ixodes aragaoi* and *Ixodes pararicinus* are frequent parasites of cattle. Other species are found on bovines more rarely as is the case of *Amblyomma aureolatum*, *Amblyomma dissimile*, *Amblyomma dubitatum*, *Amblyomma hadanii*, *Amblyomma pseudoconcolor*, *Amblyomma tigrinum*, *Dermacentor nitens*, *Ixodes longiscutatus*, *Haemaphysalis juxtakochi* [42,43,44,45]. It would be of great importance to distinguish among occasional cattle infestations with indigenous ticks from those more frequent and relevant for bovines. For example, it was already noted that, within their geographical distribution, species such as *A. neumanii* and *A. mixtum* can maintain their life cycle on bovines [42,46]. Other tick species, however, rely on wild hosts to maintain their life cycle and parasitize bovines fortuitously.

Exposure of cattle to wildlife ticks could be better understood if infestations of natural environment and indigenous hosts were also known. It is necessary to consider that environmental changing is an ongoing but uneven process throughout the Neotropics and tick ecology at the cattle-wildlife interface will necessarily acquire new features.

## 5. Anti-Tick Vaccines: An Efficacious and Sustainable Intervention for the Control of Cattle Tick Infestations

Anti-tick vaccines point to a completely different strategy to control tick infestations and pathogen transmission. It is based on the vaccination of cattle using different antigens with different roles in the physiology of ticks. The vaccines do not kill the ticks in the same way the acaricides do, and it is necessary to instruct the producer about how to observe a basic set of rules in the protection of cattle against ticks and the correct use of the different available methods for this control. Anti-tick vaccines became available in the early 1990s for the control of *R. microplus* cattle tick infestations in a cost-effective manner to reduce the use of acaricides and the selection of acaricide-resistant ticks and contamination of the environment and animal products with pesticide residues [47,48,49,50]. Vaccines using tick BM86 and SUB antigens were also used in pen trials to reduce *R. microplus* tick infestations in wildlife hosts [51]. Field trials have not been conducted with wildlife in the Neotropics, but the efficacy of the vaccine was demonstrated in red deer under field conditions in Spain [51].

Despite recent advances in the identification and characterization of tick protective antigens [52,53,54], the major challenges faced to further advance the implementation of effective vaccination strategies for the control of cattle tick infestations and tick-borne diseases include: (a) rational and effective combination of anti-tick vaccines with acaricides and other traditional control measures; (b) development and implementation of cost-effective and safe vaccines reducing infestations by multiple tick species in different hosts; (c) vaccine formulations to reduce tick infestations and pathogen infection and/or transmission; and (d) funding and fulfilling regulatory requirements for vaccine registration. To address these challenges we propose (a) to use information on tick life cycle and the effect of biotic and abiotic factors for the effective combination of multiple control measures including vaccines for the control of tick infestations and pathogen infection and transmission [55]; (b) modeling the vaccination strategies against ticks and transmitted pathogens to guide the selection of appropriate antigen combinations, target hosts and vaccination time schedule [53]; (c) to use latest omics technologies in a vaccinomics approach combined with systems biology and big data machine learning algorithms to identify new protective antigens and advance quantum immunology [56,57]; (d) to combine tick-derived and pathogen derived antigens in effective vaccine delivery formulations to target multiple tick species in domestic and both domestic and wild hosts [55,56,57,58]; (e) to develop country and host/tick species driven strategies to increase the efficacy of vaccination and other control strategies for cattle ticks and transmitted pathogens [59]. Finally, it is important to advance research on areas such as sequencing and assembly of tick genomes, vector competence, functionality of tick microbiota, functional analysis of tick-host-pathogen interactions, and pathogen control of tick/host epigenetics to develop vaccines and methods to manipulate tick genetics and microbiota for new effective interventions to control tick infestations and transmitted pathogens affecting both human and animal health [60,61].

## 6. Conclusions and Future Actions

The multi-disciplinary approaches for tick control addressed in the meeting identified several points for action, envisaged to be developed in the near future. These points were identified as in need of urgent development and include: (i) an adequate understanding of the effects of regional climate patterns on the parasitic load by ticks; (ii) the contribution of the local wild fauna to the spread of the ticks and its contribution to the support of foci of pathogens; (iii) the impact of human actions on the changing landscape patterns, configuring the availability of hosts for ticks; (iv) the development of new anti-tick vaccines effective against multiple ticks species; and (v) the development of guidelines about the importance of the correct use of acaricides and the improvements in tick control derived from the combined use of vaccination strategies and classic acaricides aimed to distribute the information to national animal health authorities. The discussions of the group also addressed the need of building a suitable modelling environment aimed at developing adequate control strategies according to the regional parameters of tick loads, the elasticity of the tick ecology adapted to the governing weather features, the prevalence of pathogens, landscape patterns, and a complete analysis of costs and benefits. These points will be addressed and reinforced in future meetings and actions within the CYTED “LaGar” project.
